# The influence of social capital on college students’ academic adaptation: the mediating role of positive emotional experience

**DOI:** 10.3389/fpsyg.2025.1670147

**Published:** 2025-12-04

**Authors:** Qingquan Tong, Jianshan Cheng, Yanxuan Yang

**Affiliations:** 1 Department of Foreign Languages, Changzhou University Huaide College, Jingjiang, China; 2 School of Foreign Languages, Wuhan Institute of Technology, Wuhan, China; 3 School of Public Administration, Central China Normal University, Wuhan, China

**Keywords:** social capital, positive emotional experience, academic adaptation, mediation, college students

## Abstract

**Introduction:**

This study aims to examine the impact of social capital on college students’ academic adaptation and to explore the underlying mechanism—specifically, the mediating role of positive emotional experience. By integrating perspectives from social capital theory and positive psychology, it seeks to enrich theoretical understandings of higher-education adaptation.

**Methods:**

A total of 428 undergraduates from a comprehensive university participated in this study. Data were gathered using a self-developed Social Capital Scale, an Academic Adaptation Scale, and a Positive Emotional Experience Scale. Descriptive statistics and confirmatory factor analysis were conducted in SPSS 26.0 and AMOS 24.0, respectively. Structural equation modeling was used to test the hypothesized paths, and the mediating effect of positive emotional experience was assessed via Hayes’s PROCESS macro (Model 4) with 5,000 bootstrap samples.

**Results:**

Social capital had a significant direct predictive effect on academic adaptation (*β* = 0.251, *p* < 0.001). The path from social capital to positive emotional experience was also significant (*β* = 0.679, *p* < 0.001), as was the path from positive emotional experience to academic adaptation (*β* = 0.583, *p* < 0.001). Bootstrap analysis indicated an indirect effect of 0.396 (95% CI [0.283, 0.523]), excluding zero, demonstrating that positive emotional experience partially mediates the relationship between social capital and academic adaptation.

**Discussion:**

These findings support social capital theory and broaden-and-build theory, revealing a synergistic mechanism whereby external social support resources enhance students’ positive emotional experiences, which in turn promote academic adaptation. Practically, universities should foster strong teacher–student and peer support networks while implementing interventions to cultivate positive emotions, thereby leveraging both pathways to improve students’ adaptation to academic life.

## 1 Introduction

With the widespread popularization of higher education, college students’ ability to adapt to complex learning tasks, role transitions, and interpersonal relationships has garnered increasing attention. Academic adaptation is commonly defined as students’ capacity to cope with academic stress, accomplish learning tasks, and achieve academic goals (Baker and Siryk, 1984). Good academic adaptation is not only closely related to academic performance but also affects students’ mental health and social functioning (Silva et al., 2025). Given the prevalence of negative psychological problems such as anxiety and burnout among today’s undergraduates, there is an urgent need for interventions rooted in positive resources (Liao et al., 2025).

Among the various beneficial resources, social capital is widely recognized as a critical external condition for supporting college students’ development. Coleman (1988) defined social capital as resources embedded within social structures—such as trust, norms, and networks—that enhance individuals’ capacity for action by facilitating cooperative behavior. Putnam (2000) further characterized social capital as the combination of social networks, mutual trust, and shared norms, constituting the driving force behind social cooperation. In educational contexts, research has shown that social capital can strengthen students’ connections with family, peers, and teachers, thereby enhancing learning motivation and sense of belonging (Wang and Eccles, 2012; Song et al., 2011). For example, positive peer relationships and robust teacher–student interaction networks can provide informational, emotional, and normative support, alleviating academic stress and promoting adaptation (Toyon, 2024).

From the perspective of positive psychology, individuals’ positive emotional states are likewise key resources for facilitating adaptation. Fredrickson's (2001, 2004) broaden-and-build theory posits that positive emotions—such as joy, hope, and gratitude—broaden individuals’ cognitive and behavioral repertoires and build enduring personal, social, and academic resources. These emotions can enhance undergraduates’ self-efficacy, stress regulation, and motivation, thus improving academic adaptation (Carmona-Halty et al., 2024). Importantly, social capital may not only function as an external structural resource but also influence adaptation by enriching individuals’ positive emotional experiences (Ma, 2021).

Although prior studies have explored student adaptation from the standpoint of social support or psychological capital, few have systematically examined the mechanism by which social capital affects academic adaptation, especially through the lens of positive emotional experience. Most existing research focuses on the direct effects of external factors such as social support, with insufficient attention to internal positive psychological processes. Constructing and testing a mediation model of social capital → positive emotional experience → academic adaptation can enrich adaptation theory and offer practical guidance: understanding the positive psychological mechanisms underlying students’ successful adaptation can inform the development of social relationship structures and mental health education in universities.

Therefore, this study investigates whether and how social capital influences college students’ academic adaptation through positive emotional experience. Specifically, it addresses the following questions: (1) Does social capital significantly promote academic adaptation? (2) What role does positive emotional experience play between social capital and academic adaptation? We thus propose the following hypotheses:

*H1*: Social capital is positively related to academic adaptation.

*H2*: Social capital is positively related to positive emotional experience.

*H3*: Positive emotional experience is positively related to academic adaptation.

*H4*: Positive emotional experience mediates the relationship between social capital and academic adaptation.

## 2 Literature review

### 2.1 Social capital and academic adaptation

Social capital refers to the resources and support individuals obtain through their social networks, including emotional bonds, information sharing, and trust relationships (Coleman, 1988). Scholars identify key components of social capital—network ties, norms of reciprocity, and trust—with Coleman particularly emphasizing its role in facilitating cooperation, establishing trust, and creating shared norms. In educational contexts, research has shown that social capital provides college students with learning resources, informational support, and emotional encouragement, thereby promoting academic adaptation (Song et al., 2011). In this study, we conceptualize social capital as a multidimensional construct comprising (a) perceived peer support—emotional and instrumental assistance from classmates and friends; (b) perceived faculty support—academic guidance, mentoring, and feedback from instructors; and (c) institutional resources—access to counseling, tutoring, learning spaces, and formal student services (Coleman, 1988; Putnam, 2000; Bourdieu, 1986). Prior empirical work indicates that perceived social support robustly predicts psychological resilience and successful academic adjustment (Cohen and Wills, 1985; Ozbay et al., 2007; Wilcox et al., 2005), which motivates our decision to analyze each dimension separately. Theoretically, we link these distinctions to Fredrickson’s broaden-and-build theory (Fredrickson, 2001): social capital fosters positive emotions—through experiences of belonging, competence, and safety—that broaden students’ thought–action repertoires and, over time, build durable coping resources that facilitate adaptation and academic engagement. Consistent with Tripon’s (2023) discussion of emotional competencies in STEM contexts, we further posit that emotional competence and regulation may mediate or moderate the pathways from social capital to resilience and adjustment, suggesting an integrative model in which interpersonal and institutional supports operate partly through enhanced positive affect and emotion regulation. Both family social capital (e.g., parental support and expectations) and school social capital (e.g., relationships with teachers and peers) have been found to be significantly positively correlated with students’ academic performance and adaptation levels (Wang and Eccles, 2012). Within the university environment, social capital manifests as interactive networks among students, families, peers, and teachers. Its primary function is to facilitate better adaptation to academic and social environments by providing emotional support, academic resources, and behavioral norms (Fredrickson, 2001; Toyon, 2024). Recent studies suggest that students possessing strong social capital are better able to utilize educational opportunities and cope with academic stress, thus improving their academic adaptation (Hassan et al., 2023). Specifically, those with more campus-based social ties—such as active involvement in clubs or strong teacher-student relationships—are more likely to access information, receive support, and experience a sense of belonging when facing academic challenges (Wang and Eccles, 2012). Overall, social capital on campus serves as a foundational external support by offering informational and psychological resources that help students effectively cope with academic stress and social adjustment, thus enhancing their academic success. This underscores social capital as a key variable in constructing a supportive learning ecology in higher education.

### 2.2 Positive emotional experience and academic adaptation

Positive emotions refer to the favorable psychological states that individuals experience in learning and life, such as joy, satisfaction, pride, and hope (Fredrickson, 2001). According to Fredrickson’s “broaden-and-build theory,” positive emotions broaden individuals’ cognitive and behavioral repertoires, thereby building lasting personal resources—including psychological and social resources—that enhance coping and adaptability (Fredrickson, 2004). In academic contexts, research has shown that positive emotions are significant antecedents of students’ learning processes, academic achievement, and psychological well-being. Within college settings, positive emotions not only strengthen learning motivation but also increase student engagement in both academic and social contexts (Carmona-Halty et al., 2024). Cobo-Rendón et al. (2023) further found that positive academic emotions significantly predict college students’ overall adaptation to coursework, peers, and campus culture. Additionally, positive emotions can buffer academic stress and reduce the impact of negative affect, thereby helping to maintain psychological balance and learning motivation (Lyubomirsky et al., 2005). In this process, positive emotion plays a “bridging” role: it links external social support with internal experiences and drives students to develop positive learning attitudes and behaviors. In summary, based on the broaden-and-build theory, positive emotions enrich students’ cognitive and social resources, enhancing their psychological adjustment and playing a constructive role in academic adaptation.

### 2.3 Concept, current research, and influencing factors of academic adaptation

Academic adaptation is the process through which college students continuously adjust and optimize their cognitive, emotional, and behavioral responses in higher-education settings, manifesting in areas such as learning engagement, self-efficacy, emotional regulation, and stress coping (Baker and Siryk, 1984). Studies indicate that effective academic adaptation is closely linked to students’ academic performance and retention, and also has a significant impact on their psychological health (Silva et al., 2025). Among the many factors influencing academic adaptation, psychological capital is considered one of the core predictors. Psychological capital—comprising self-efficacy, hope, resilience, and optimism—helps students cope with academic pressure more proactively, thereby enhancing their level of academic adjustment. For example, Yang and Zhang (2015) found that college students’ positive psychological capital is significantly negatively correlated with academic burnout and serves as a significant predictor. Meng and Yang (2012) further noted that psychological capital moderates the relationships between academic stress, psychological anxiety, and subjective well-being. Beyond personal psychological resources, family capital also significantly affects academic adaptation. Zhang et al. (2022) found that family cultural capital significantly promotes college students’ learning engagement; research by Mi et al. (2018) showed that family economic and cultural capital affect students’ process-level learning inputs such as time, effort, attention, and also influence the development of active learning habits. Thus, both internal psychological capital and external family capital serve as important drivers in enhancing students’ academic adjustment. Additionally, social support is recognized as another vital resource: support from peers, family, and the school can enhance psychological capital and thus improve adaptation (Carmona-Halty et al., 2024). Hassan et al. (2023) noted that social support can reduce students’ anxiety and loneliness, and promote academic adjustment by strengthening psychological capital. Moreover, individual traits—such as social skills, problem-solving ability, self-monitoring, and coping strategies—are also strongly linked to academic adaptation (Silva et al., 2025). Research by Silva et al. (2025) indicates that higher levels of physical and mental health (quality of life), geographic proximity to campus, and receiving scholarships are positive predictors of academic adaptation, whereas lack of exercise has a negative effect. Overall, academic adaptation is influenced by multiple factors, including students’ psychological and physical state, social support networks, academic capability, and school environment (Hassan et al., 2023). To support students’ successful transition to university life, institutions should focus on strengthening students’ psychological capital, enhancing social support, and improving learning environments.

In academic contexts, positive emotions help enhance learning motivation, improve memory and attention, and increase engagement with academic tasks (Pekrun et al., 2002). Social capital provides support and a sense of belonging, which often enhances students’ psychological security and self-efficacy, thereby increasing positive emotional experience (Ma, 2021). Empirical evidence shows that social support is positively correlated with students’ well-being and learning satisfaction, and that high levels of positive emotion are closely associated with academic adaptation and academic achievement (King et al., 2015). Therefore, social capital may promote academic adaptation by enhancing students’ positive emotional experience—that is, positive emotional experience functions as a mediator between the two variables.

## 3 Methods

### 3.1 Participants

The sample was drawn from undergraduates enrolled at a single comprehensive university in China, yielding 428 valid respondents after data screening. The sample comprised 172 males (40.2%) and 256 females (59.8%), with ages ranging from 18 to 23 years (M = 20.5). Participants represented a broad range of majors (e.g., Accounting, Chemical Engineering, Materials Science, Law, Marketing) and three academic levels: freshmen (*n* = 168, 39.2%), sophomores (*n* = 123, 28.7%), and juniors (*n* = 137, 32.0%), providing reasonable disciplinary and year-level coverage of the undergraduate population at the institution. Data were collected via an online questionnaire Wenjuanxing on a voluntary basis; responses were excluded if completion times fell below prespecified thresholds or if a single response option was selected at an anomalously high rate. After these quality-control procedures, 428 completed questionnaires remained for analysis. The study protocol was approved by the university ethics committee and all participants provided informed consent prior to participation.

### 3.2 Measures

#### 3.2.1 Social Capital Scale

We developed a 15-item Social Capital Scale for undergraduates, theoretically grounded in Coleman (1988) and Putnam (2000) and organized into three subscales: Family Social Capital, Peer Support, and Student–Teacher Trust. Items are rated on a 5-point Likert scale (1 = Strongly disagree to 5 = Strongly agree), with higher scores indicating greater social capital. The Scale was administered in Chinese and several items were reverse-worded (clearly marked on the questionnaire) and were reverse-scored during data entry. In a pilot test the overall scale demonstrated excellent internal consistency (Cronbach’s *α* = 0.92). In the full study sample each subscale likewise showed high internal consistency (Family α = 0.89; Peer Support α = 0.90; Student–Teacher Trust α = 0.88), and composite reliabilities exceeded 0.85 while average variances extracted were greater than 0.50, supporting convergent validity. Factorial validity was evaluated using confirmatory factor analysis in AMOS 26.0: the hypothesized three-factor model provided good fit to the data (CMIN/DF ≈ 1.9, *p* < 0.001; GFI ≈ 0.94; AGFI ≈ 0.91; CFI ≈ 0.98; RMSEA ≈ 0.04; RMR ≈ 0.03). Subscale inter-correlations were moderate to strong and in the expected direction (Family–Peer r ≈ 0.54; Family–Teacher r ≈ 0.46; Peer–Teacher r ≈ 0.61; all *p* < 0.001), indicating related but distinct dimensions. Taken together, these results indicate that the Social Capital Scale exhibits satisfactory reliability and factorial validity.

#### 3.2.2 Positive Affect Scale

The Positive Affect Scale used in this study was developed from Fredrickson’s broaden-and-build theory and the positive subscale of the Modified Differential Emotions Scale (mDES) (Fredrickson, 2001). Ten prototypical positive emotions were selected (joy, contentment, interest, pride, inspiration, hope, amusement, gratitude, awe, and love) and items were adapted to the campus context to form a 10-item scale. Respondents rated each item on a 5-point frequency scale (1 = Almost never, 2 = A little, 3 = Moderately, 4 = Quite a lot, 5 = Extremely), referring to the past two weeks; the overall positive affect score is the mean of the ten items, with higher scores indicating stronger recent positive affect. In pilot testing the scale showed high internal consistency (Cronbach’s *α* = 0.93), which compares favorably with reported reliabilities for the mDES positive subscale (α ≈ 0.79–0.85) and indicates good coherence for the campus-adapted items. Structural validity was examined with confirmatory factor analysis: the one-factor model demonstrated excellent fit (CMIN/DF = 2.453; GFI = 0.912; CFI = 0.925; RMSEA = 0.065), supporting the unidimensional structure of the adapted positive affect measure. These psychometric results indicate that the Positive Affect Scale is a reliable and valid instrument for assessing students’ recent positive emotional experiences.

#### 3.2.3 Academic Adjustment Scale

The 12-item Academic Adjustment Scale used in this study was developed by adapting items from established instruments. Specifically, items for the Learning Engagement dimension were adapted from the Utrecht Work Engagement Scale — Student version (UWES-S), which operationalizes engagement as vigor, dedication, and absorption in student populations (Carmona-Halty et al., 2019). Items for Academic Self-Efficacy were informed by Chemers et al.’s (2001) work on academic self-efficacy and first-year college student performance and adjustment. Items for Academic Stress Coping drew on established coping inventories—principally Carver’s Brief COPE and Folkman and Lazarus’s Ways of Coping—to capture problem-focused and emotion-regulation strategies (Carver, 1997). Items employ the same 5-point Likert response format used for the Social Capital Scale (1 = Strongly disagree … 5 = Strongly agree), with higher scores indicating better academic adjustment. The Scale was administered in Chinese and several items were phrased in the opposite direction (these were clearly labeled on the questionnaire) and were reverse-scored during data entry and cleaning (e.g., 1 → 5, 2 → 4) to ensure consistent scoring. The Learning Engagement dimension assesses students’ vigor, dedication, and sustained involvement in coursework and learning activities; the Academic Self-Efficacy dimension measures confidence in one’s ability to master academic tasks and succeed in examinations and assignments; and the Academic Stress Coping dimension evaluates the frequency and effectiveness of problem-focused strategies and emotion-regulation techniques used when facing academic stressors. In a preliminary survey (*N* = 200) the overall scale demonstrated excellent internal consistency (Cronbach’s *α* = 0.95). Factorial validity was supported by confirmatory factor analysis (three-factor model): CMIN = 217.643, df = 52, *p* < 0.001; CMIN/DF = 2.785; GFI = 0.955; AGFI = 0.882; RMR = 0.048, indicating an acceptable to good fit. Subscale inter-correlations were moderate to strong and in theoretically expected directions: Learning Engagement — Academic Self-Efficacy *r* = 0.62, *p* < 0.001; Learning Engagement — Academic Stress Coping *r* = 0.49, *p* < 0.001; Academic Self-Efficacy — Academic Stress Coping *r* = 0.56, *p* < 0.001. Collectively, these results indicate that the Academic Adjustment Scale exhibits high internal consistency, satisfactory convergent validity, and a coherent three-factor structure.

### 3.3 Data analysis

Using data from 428 undergraduates, structural equation modeling (SEM) was conducted to test the direct effect of social capital on academic adjustment and to examine the mediating role of positive affect via moderation–mediation analyses. Data were processed in SPSS 26.0 and AMOS 24.0, with mediation tested using Hayes’ (2013) PROCESS macro (Model 4) with 5,000 bootstrap samples to ensure robustness and accurate effect estimation.

#### 3.3.1 Common method bias test

To assess potential common method bias, we applied Harman’s single-factor test via exploratory factor analysis (EFA) on 37 self-reported variables (covering family support, peer interaction, teacher relations, emotional experience, learning ability, etc.). Principal component analysis without rotation was performed in SPSS 26.0, extracting factors with eigenvalues > 1, and the Kaiser–Meyer–Olkin (KMO) and Bartlett’s tests confirmed data suitability. Results showed KMO = 0.938 (> 0.70) and Bartlett’s χ^2^ = 11,227.420 (df = 1,891, *p* < 0.001), indicating strong intercorrelations suitable for factor analysis (Table 1).

**Table 1:** Confirmatory factor analysis.

Index	Result	Interpretation
KMO value	0.938	Highly suitable for factor analysis
Bartlett X^2^	11227.420 (*p*<0.001)	Significant correlations exist among variables

#### 3.3.2 Normality tests

Prior to hypothesis testing, we conducted Kolmogorov–Smirnov (with Lilliefors correction) and Shapiro–Wilk tests on the three primary variables (Social Capital, Positive Affect, Academic Adjustment). All tests were non-significant (*p* > 0.05), and skewness and kurtosis values fell within ±1, indicating approximate normal distributions suitable for subsequent parametric analyses. The Kolmogorov–Smirnov statistics were non-significant at the 0.05 level, and Shapiro–Wilk W values approached 1. Thus, regression analyses and PROCESS-based mediation and moderation tests proceeded under the assumption of normality (Table 2).

**Table 2:** Results of normality test.

Variable	Kolmogorov–Smirnov		Shapiro–Wilk		Skewness	Kurtosis
	Statistic	*p*-value	W	*p*-value	(SE)	(SE)
Social capital	0.081	0.084	0.992	0.123	0.12	−0.08
Positive affect	0.074	0.112	0.994	0.142	0.10	−0.05
Academic adjustment	0.092	0.056	0.989	0.085	−0.08	0.15

Both skewness and kurtosis are calculated with standard error (SE) as the denominator; *p* > 0.05 indicates that the normality assumption is not rejected.

### 3.4 Descriptive statistics analyses

#### 3.4.1 Gender differences

Data were analyzed in SPSS 26.0. First, Levene’s test examined the homogeneity of variances; then independent-samples t-tests compared mean scores between male and female students, with significance set at *p* < 0.05. Results indicated that on the Social Capital Scale, males had a mean of 3.69 (SD = 0.69) versus females’ mean of 3.49 (SD = 0.70), t (192) = 1.95, *p* = 0.053 (> 0.05), showing no statistically significant difference. Although males trended slightly higher across subscales, none reached significance. For overall Positive Affect, males’ mean was 3.89 (SD = 0.63) and females’ 3.73 (SD = 0.67), t = 1.65, *p* = 0.102 (> 0.05), and all sub-emotion comparisons (e.g., joy, contentment, interest) likewise yielded *p* > 0.05. Thus, no significant gender differences emerged in strength or type of positive emotions. On the Academic Adjustment Scale, males averaged 3.73 (SD = 0.67) and females 3.61 (SD = 0.69), *t* = 1.19, *p* = 0.235 (> 0.05), with no significant differences across its dimensions—likely reflecting a fair educational environment (Table 3).

**Table 3:** Gender differences analysis.

Variable	Mean (SD) of male	Mean (SD) of female	t-value	*p*-value	95% CI lower limit	95% CI upper limit
Social capital	3.69 (0.69)	3.49 (0.70)	1.95	0.053	−0.002	0.399
Positive affect	3.89 (0.63)	3.73 (0.67)	1.65	0.102	−0.031	0.347
Academic adjustment	3.73 (0.67)	3.61 (0.69)	1.19	0.235	−0.078	0.316

#### 3.4.2 Grade-level differences

Grade level (freshman/sophomore/junior) served as the independent variable in one-way ANOVAs on total Social Capital, Positive Affect, and each Academic Adjustment dimension. Levene’s tests confirmed homogeneity of variances for Social Capital (*F* = 1.742, *p* = 0.178), Positive Affect (*F* = 0.429, *p* = 0.651), Learning Engagement (*F* = 0.982, *p* = 0.374), Academic Self-Efficacy (*F* = 1.215, *p* = 0.297), and Stress Coping (*F* = 0.156, *p* = 0.856), justifying ANOVA. Significance thresholds were set at *p* < 0.10 (marginal) and *p* < 0.05 (significant).

ANOVA results showed a marginal grade-level effect on Social Capital total score, *F* = 2.483, *p* = 0.086. *Post hoc* comparisons (Bonferroni/Games–Howell) indicated sophomores (*M* = 3.755) scored marginally higher than juniors (*p* = 0.091). This may reflect sophomores’ heightened engagement in clubs and practical activities, which bolster social resources, whereas juniors face internship and course pressures that reduce social participation. Positive Affect means were similar across grades (freshmen M = 3.783; sophomores *M* = 3.864; juniors M = 3.747), *F* = 0.304, *p* = 0.738, indicating stability of positive emotion throughout the undergraduate years—perhaps due to ongoing faculty and peer support. For the three Academic Adjustment dimensions—Learning Engagement (*F* = 1.321), Self-Efficacy (*F* = 1.836), and Stress Coping (*F* = 0.211)—all *p* > 0.10, showing no significant grade-level differences. Although sophomores scored slightly higher in engagement (*M* = 3.764) and self-efficacy (*M* = 3.702), these differences were not significant, suggesting stable academic motivation, self-efficacy, and coping skills from freshman to junior year (Table 4).

**Table 4:** Grade-level differences analysis.

Scale/dimension	F-value	*p*-value	Comparison of marginally significant differences by grade (Bonferroni)
Family social capital	2.804	0.063	Sophomore > Junior (*p* = 0.078)†
Peer support	1.834	0.142	Not significant
Teacher - student trust	1.335	0.264	Not significant
Total score of social capital	2.483	0.086	Sophomore > Junior (p = 0.091)†
Positive emotional experience	0.304	0.738	Not significant
Learning engagement	1.321	0.269	Not significant
Self-efficacy	1.836	0.162	Not significant
Coping with academic pressure	0.211	0.810	Not significant
Total score of academic adaptation	0.872	0.457	Not significant

^†^Denotes marginal significance (*p* < 0.10); all dimensions passed Levene’s test (*p* > 0.05) prior to ANOVA.

### 3.5 Multiple regression analysis

The regression model yielded a multiple correlation coefficient *R* = 0.888, *R*^2^ = 0.789, and adjusted *R*^2^ = 0.786, indicating that social capital, positive affect, and psychological resilience collectively explained 78.9% of the variance in academic adjustment. The small difference between *R*^2^ and adjusted *R*^2^ suggests a well-fitted model given the variable count and sample size. ANOVA for the regression showed *F* (3, 190) = 237.253, *p* < 0.001, confirming the model’s overall significance compared to a null model (Table 5).

**Table 5:** Overall model fit.

Indicator	Value	Explanation
R	0.888	Multiple correlation coefficient, indicating the overall correlation between the predictor variable and academic adaptation
R^2^	0.789	Coefficient of determination, indicating that 78.9% of the variation in academic adaptation can be jointly explained by the three variables
Adjusted R^2^	0.786	Modified R^2^ after considering the number of variables, indicating a robust model fit
Standard Error	0.316	The model has a small prediction error and a good fit
F-value	237.253	The overall model equation is significant
Significance	<0.001	The regression model is overall significant and statistically meaningful

Standardized Beta coefficients indicated both predictors had significant positive effects on academic adjustment at *p* < 0.001: Social Capital (*β* = 0.251, *t* = 4.930) and Positive Affect (*β* = 0.372, *t* = 6.681). Thus, a one-standard-deviation increase in social capital corresponds to a 0.251 SD increase in adjustment, and a similar increase in positive affect relates to a 0.372 SD rise—suggesting a stronger effect of emotions. Variance inflation factors (VIF < 5) and tolerances (> 0.1) indicate no collinearity issues. Residual analyses showed all standardized residuals within ±3 and approximately normal distribution, supporting model adequacy (Table 6).

**Table 6:** Regression coefficients for predictors of academic adjustment.

Independent variable	B (unstandardized coefficient)	Beta (standardized coefficient)	t-value	Significance	VIF
Social Capital	0.245	0.251	4.930	<0.001	2.334
Positive Emotional Experience	0.386	0.372	6.681	<0.001	2.792

All variables have a positive impact on academic adaptation, and there is no multicollinearity issue (VIF < 5, tolerance > 0.1).

### 3.6 Mediation analysis

Using PROCESS v4.2_beta (Model 4), we tested whether positive affect (PE) mediates the effect of social capital (SC) on academic adjustment (AA), controlling for gender, grade level, and family economic status. With 5,000 bootstrap samples, mediation is significant if the 95% CI excludes zero. Results indicated that SC predicted PE (path a: coefficient = 0.6788, SE = 0.0477, *t* = 14.2195, *p* < 0.001) and PE predicted AA (path b: coefficient = 0.5834, SE = 0.0557, *t* = 10.4712, *p* < 0.001). The direct effect of SC on AA, controlling for PE (path c′), remained significant (coefficient = 0.3579, SE = 0.0527, *t* = 6.7957, *p* < 0.001) (Table 7).

**Table 7:** Mediation path coefficients (a, b, and c′).

Model	Path	β	SE	*t*	*p*	95% BCa CI
PE regression model	SC → PE	0.6788	0.0477	14.22	<0.001	[0.5846, 0.7730]
AA regression model (with mediating variable)	PE → AA	0.5834	0.0557	10.47	<0.001	[0.4735, 0.6933]
AA regression model (with mediating variable)	SC → AA(c’)	0.3579	0.0527	6.80	<0.001	[0.2540, 0.4618]

The indirect effect (SC→PE→AA) was 0.3960 (BootSE = 0.0607), 95% CI = [0.2828, 0.5230], excluding zero—indicating a significant partial mediation. Control variables had non-significant effects on PE and AA (*p* > 0.05). In sum, positive affect partially mediates the relationship between social capital and academic adjustment: social capital both directly enhances adjustment and indirectly facilitates it by boosting positive emotions (Table 8).

**Table 8:** Direct effect, indirect effect and total effect.

Effect type	Path	Coefficient	BootSE	95% BCa CI
Direct effect (c’)	SC → AA	0.3579	0.0527	[0.2540, 0.4618]
Indirect effect (a × b)	SC → PE → AA	0.3960	0.0607	[0.2828, 0.5230]
Total effect (c)	SC → AA	0.7539	–	–

## 4 Discussion

This study found that undergraduate students’ social capital significantly enhances academic adjustment, and this effect is partially mediated by increased positive affect. The finding is theoretically important: it confirms Coleman's (1988) notion that social capital provides individuals with information and support through interpersonal networks, while also aligning with Fredrickson’s Broaden-and-Build theory (2001, 2004). From a positive psychology perspective, abundant social connections offer emotional support and resources, strengthening students’ sense of belonging and self-efficacy (Song et al., 2011; Wang and Eccles, 2012), thereby alleviating academic stress. Concurrently, supportive interactions make students more likely to experience joy, contentment, and other positive emotions (Ma, 2021), which in turn broaden their cognitive–behavioral repertoire, building psychological resilience and learning motivation (Fredrickson, 2004). Thus, our findings elucidate a mechanism in which external social-environmental resources and internal psychological resources jointly promote academic adjustment: social capital forms the external resource base for students facing academic challenges, whereas positive affect helps convert those resources into positive learning behaviors and emotional energy, together fostering academic adjustment. In short, social capital can supply opportunities and support that increase students’ experiences of joy, interest, and contentment; these positive affects then broaden students’ cognitive and behavioral repertoires in ways that promote academic adjustment.

Comparatively, our results are consistent with and extend previous domestic and international research. Liao et al. (2025), for example, also found that perceived social support significantly facilitates student adjustment, though they focused on basic psychological need fulfillment as the mediator—our study expands this by examining positive emotions. Similarly, Carmona-Halty et al. (2019) found that student positive affect promotes academic achievement by enhancing psychological capital and learning engagement, which aligns with our observed pathway of positive affect enhancing academic adjustment. Hassan et al. (2023) found that perceived social support and positive psychological capital positively predict both academic adjustment and success; this dovetails with our finding that social capital positively correlates with academic adjustment and partially operates through positive affect. Moreover, Cobo-Rendón et al. (2023) showed that achievement-related positive emotions predict university students’ adjustment and continued learning intention, supporting the pathway we observed. Castro Torres et al. (2022) claimed that Bonding social capital (close ties) reduces academic stress and psychological symptoms through intermediate psychosocial mechanisms (e.g., coping resources), supporting multi-step mediation. These serial/parallel mediation results mirror your partial mediation finding and show that social capital can operate via psychological pathways (including emotions/coping resources) to affect academic strain/adaptation. Dong et al. (2023) found that Social media–based interactions increased bonding social capital, which in turn improved learning engagement and adjustment indicators. It shows another route by which social capital (including digitally mediated ties) supports engagement—consistent with your focus on social ties as an external resource that leads to better academic functioning (often via psychosocial intermediaries).

However, previous research on gender and grade-level differences in student academic adjustment has yielded mixed results. Zhang et al. (2014) found that second- and third-year students exhibit the lowest academic adjustment, with a dip and then rebound trend. Wu and Zhang (2023), in a survey of 311 undergraduates, reported self-esteem significantly predicted adjustment, and peer relationships partially mediated this effect—with full mediation in males but not females. In Macau, Chan and Jheng (2020) found that female students outperformed males in academic adjustment, intimate relationships, and value adaptation, with no grade-level differences. Our results do not contradict these studies; differences stem primarily from sample characteristics, measurement dimensions, and educational context. Our sample was drawn from a single comprehensive university, focusing only on learning engagement, self-efficacy, and stress coping—not broader constructs such as psychological health or community support. Contemporary higher education, characterized by homogeneity and equity, may diminish gender and grade disparities: in a modern comprehensive university, male and female students enjoy equal access to faculty interaction, peer communication, and resource acquisition; likewise, the “competency-based” educational paradigm may attenuate gender-based differences in learning motivation, self-efficacy, and stress coping.

Although our study shows that social capital promotes academic adjustment in part by increasing positive affect, several strands of prior research provide important qualifications and directions for interpretation. First, intervention experiments that attempted to increase social capital (e.g., network closure or tie-building programs) have produced mixed results, sometimes yielding little or no improvement in objective academic outcomes such as test scores or GPA (Gamoran et al., 2021). These mixed intervention findings suggest three cautions. First, social capital is heterogeneous: bonding ties (close friends, family) and bridging ties (broader acquaintances, institutional links) differ in their mechanisms and potency. Second, changes in subjective measures (affect, coping, engagement) are easier to achieve and detect in the short term than changes in objective academic metrics, which may require longer exposure and structural opportunity to shift. Third, artificially constructed ties in short-term interventions may not reproduce the trust, reciprocity, and embedded resources of naturally accumulated networks; therefore, null intervention effects on achievement do not invalidate evidence that social capital is important for students’ affective and behavioral adjustment.

Second, large-sample observational work shows that the returns to social capital are context-dependent, particularly on family socioeconomic status (SES) and other background variables (Chetty et al., 2022a, b; Dufur et al., 2024). When studies tightly control for SES and structural resources, the independent effect of social capital sometimes attenuates or becomes heterogeneous across subgroups. This suggests that social capital often operates alongside—and sometimes through—material and structural advantages; in more homogeneous university samples (like ours) social-capital effects on self-reported adjustment may appear stronger than they would in highly heterogeneous populations. Accounting for SES as a covariate and testing moderation by SES will therefore be important for assessing generalizability.

Third, several recent empirical works identify alternative or complementary mediators—basic psychological needs satisfaction (belonging, competence, autonomy), psychological capital (hope, efficacy, resilience, optimism), and self-regulated learning—that can explain the link between social support/capital and academic engagement or adjustment (Liao et al., 2025; Martínez-López et al., 2023; Chen et al., 2023). These variables are often intercorrelated with positive affect and may statistically absorb indirect effects depending on measurement detail and model specification. Thus, affect should be seen as one plausible conversion mechanism among several; a fuller account will model parallel and serial mediation pathways (for example, Social Capital → Need Satisfaction → Positive Affect → Adjustment) to estimate unique and shared contributions.

In practical terms, these syntheses imply that interventions aiming to improve student adaptation should combine relational-network supports with activities that cultivate durable emotional and self-regulatory resources (e.g., mentoring plus PsyCap training, or peer-coaching plus brief positive-emotion practices). Such combined approaches are more likely to convert external social resources into sustained academic adjustment than single-focus tie-building alone. Overall, the prior literature does not contradict our core finding; rather, it clarifies boundary conditions, alternative mechanisms, and design choices needed to generalize and translate the Social Capital → Positive Affect → Academic Adjustment pathway into robust theory and scalable practice.

### 4.1 Implications for practice

Based on these findings, this study offers guidance for educational practice and psychological intervention. First, universities should intentionally cultivate diversified social support networks to strengthen students’ social capital (Song et al., 2011; Hassan et al., 2023). For example, facilitating trust and communication between faculty and students, establishing peer-support systems, and encouraging participation in clubs and community service can enhance connections among students and between families and schools, thereby providing abundant learning resources and emotional support. Second, fostering and managing students’ positive affect should receive attention. Schools can implement positive psychology interventions—such as gratitude journaling, emotion-regulation training, and optimism workshops—or integrate relevant courses into the student curriculum (Huang, 2021). Such interventions boost students’ well-being and positive affect, which enhances motivation and adjustment. Additionally, support should be tailored to grade-level challenges: the sophomore year is critical for social resource accumulation and academic pressure transition—interventions like social-skills training or motivational seminars may help convert social capital into learning motivation. For juniors facing greater pressure, stress-management workshops and peer support groups could help prevent social withdrawal. Across all years, psychological counseling should include resilience and emotion-regulation content within routine programs. Overall, the study underscores the significance of both external social capital and internal psychological processes in cultivating student academic adjustment—offering empirical references to education managers and psychological practitioners to develop integrated systemic policies and interventions (Wang and Eccles, 2012; Huang, 2021).

### 4.2 Limitations and future directions

Several limitations warrant caution. First, the convenience sample of 428 undergraduates from a single university may limit generalizability—future research should broaden sampling across regions and institution types. Second, the cross-sectional design precludes causal inference regarding social capital, positive affect, and academic adjustment; longitudinal designs or intervention studies would better establish causal mechanisms. Third, the study employed self-constructed social capital and academic adjustment scales, which, though reliable in our sample, lack cross-sample validation. Self-report measures may also introduce common method bias or social desirability effects. Fourth, we did not examine other relevant variables such as socioeconomic status, personality traits, or negative life events, which may affect outcomes. Different types of social capital (e.g., bonding vs. bridging) could also have distinct influences on adjustment and should be explored in future studies.

Building on these limitations and findings, future research might: (1) adopt longitudinal or experimental designs—such as repeated measures or social-capital interventions—to test dynamic and causal effects of social capital and positive affect on academic adjustment; (2) expand the sample and cultural scope—examining model applicability in various regions, disciplines, age groups (e.g., graduate or vocational students), and conducting cross-cultural comparisons to identify contextual moderators; (3) explore additional mediators or moderators—such as academic motivation, self-esteem, attributional style, and emotion-regulation ability—to develop more comprehensive models; (4) employ multiple assessment methods for social capital and adjustment—using social network analysis, teacher/parent reports, etc.—to reduce self-report bias. Ultimately, future investigations should deepen understanding of how social capital interacts with academic adjustment in undergraduates, offering more robust theoretical and practical evidence.

## 5 Conclusion

This empirical study of 428 undergraduate students confirmed that social capital significantly enhances academic adjustment and that positive affect partially mediates this relationship. Specifically, students with greater social resources and interpersonal support experience more joy, contentment, and other positive emotions, which bolster their ability to cope with academic challenges and improve adjustment. These findings enrich theoretical research on social capital and academic adaptation, emphasizing positive affect’s mediating role in light of Fredrickson’s Broaden-and-Build theory (2004). The study’s empirical contribution lies in revealing, from an integrated synergy perspective, how external social capital and internal psychological states jointly influence academic adjustment, introducing a more explanatory theoretical framework in the adaptation domain.

Theoretically, our results show that robust social capital can be transformed into individual resources through positive emotions, thereby promoting academic adjustment—thus extending previous studies that focused solely on direct effects (Coleman, 1988; Fredrickson, 2001). Empirically, this study fills a domestic research gap on the social capital → positive emotion → adaptation relationship, providing new evidence for research on student adjustment. Practically, it suggests that when designing strategies to improve students’ adjustment capabilities, education administrators and mental health professionals should address both social-environmental optimization and positive-psychology cultivation. For example, policy measures may guide universities to enhance teacher–student communication and peer-support systems while implementing training courses in optimism and gratitude—interventions that are expected to elevate students’ motivation and adaptation (Wang and Eccles, 2012; Huang, 2021). In the future, our findings may inform targeted student support programs and psychological education plans, helping reduce academic burnout and dropout risk, and fostering healthy development in higher education.

## Data Availability

The raw data supporting the conclusions of this article will be made available by the authors, without undue reservation.
